# Chinese herbal decoction, Yi-Qi-Jian-Pi formula exerts anti-hepatic fibrosis effects in mouse models of CCl_4_-induced liver fibrosis

**DOI:** 10.1016/j.heliyon.2024.e26129

**Published:** 2024-02-22

**Authors:** Shiyan Yang, Yajun Cheng, Xiaolong Wang, Suyang Yue, Xi Wang, Li Tang, Hailun Li, Jie Zhang, Qingping Xiong, Shanzhong Tan

**Affiliations:** a Department of Integrated TCM and Western Medicine, Nanjing Hospital Affiliated to Nanjing University of Chinese Medicine, Nanjing, 210023, China; b Department of Gastroenterology, Huai'an Hospital Affiliated to Xuzhou Medical University, Huai'an, 223002, China; c Department of Gastroenterology, People's Hospital of Lianshui, Huai'an, 223000, China; d Department of General Surgery, Tumor Hospital of Huai'an, Huai'an, 223200, China; e Department of Gastroenterology, Nanjing Hospital of Chinese Medicine Affiliated to Nanjing University of Chinese Medicine, Nanjing, 210001, China; f Department of Nephrology, Huai'an Hospital Affiliated to Xuzhou Medical University, Huai'an, 223001, China; g Department of Endocrinology, Huai'an Hospital Affiliated to Xuzhou Medical University, Huai'an, 223002, China; h Jiangsu Key Laboratory of Regional Resource Exploitation and Medicinal Research, Huaiyin Institute of Technology, Huai'an, 223003, China

**Keywords:** Hepatic fibrosis, Herbal medicine, Intestinal flora homeostasis, Network pharmacology

## Abstract

**Background:**

Yi-Qi-Jian-Pi Formula (YQJPF) is a herbal medicine that is used to treat patients with liver failure. However, scientific evidence supporting the treatment of hepatic fibrosis with YQJPF has not been forthcoming. The present study aimed to determine the mechanisms underlying the anti-fibrotic effects of YQJPF in mouse models of hepatic fibrosis.

**Methods:**

Mice were randomly assigned to control, hepatic fibrosis model, silymarin (positive treated), and low-, medium- and high-dose YQJPF (7.5, 15, and 30 g/kg, respectively) groups. Liver function, inflammatory cytokines, and oxygen stress were analyzed using ELISA kits. Sections were histopathologically stained with hematoxylin-eosin, Masson trichrome, and Sirius red. Macrophage polarization was measured by flow cytometry and immunofluorescence. Potential targets of YQJPF against hepatic fibrosis were analyzed by network pharmacology of Chinese herbal compound and the effects of YQJPF on the transforming growth factor-beta (TGF-β)/Suppressor of Mothers against Decapentaplegic family member 3 (Smad3) signaling pathway were assessed using qRT-PCR and immunohistochemical staining. Finally, metagenomics and LC-MS/MS were used to detect the intestinal flora and metabolites of the mice, and an in-depth correlation analysis was performed by spearman correlation analysis. The data were compared by one-way ANOVA and least significant differences (LSDs) or ANOVA-Dunnett's T3 method used when no homogeneity was detected.

**Results:**

We induced hepatic fibrosis using CCl_4_ to establish mouse models and found that YQJPF dose-dependently increased body weight, improved liver function, and reversed hepatic fibrosis. Elevated levels of the pro-inflammatory factors IL-1β, IL-6, and TNF-α in the model mice were substantially decreased by YQJPF, particularly at the highest dose. Levels of serum malondialdehyde and superoxide dismutase (SOD) activity were elevated and reduced, respectively. The malondialdehyde concentration decreased and SOD activity increased in the high-dose group. M1 polarized macrophages (CD86) in the mouse models were significantly decreased and M2 polarization was mildly decreased without significance. However, high-dose YQJPF increased the numbers of M2 macrophages and inhibited TGF-β/Smad3 signaling. Metagenomic and non-targeted metabolomics detection results showed that YQJPF could regulate intestinal homeostasis, and Spearman correlation analysis showed that the abundance of Calditerrivibrio_nitroreducens was significantly negatively correlated with 18β-glycyrrhetinic acid. It is suggested that Calditerrivibrio_nitroreducens may reduce the anti-fibrosis effect of licorice and other Chinese herbs by digesting 18β-glycyrrhetinic acid.

**Conclusions:**

YQJPF can reverse liver fibrosis by inhibiting inflammation, suppressing oxidative stress, regulating the immunological response initiated by macrophages, inhibiting TGF-β/Smad3 signaling and regulating intestinal flora homeostasis. Therefore, YQJPF may be included in clinical regimens to treat hepatic fibrosis.

## 1 Introduction

Hepatic fibrosis is prevalent worldwide and the main causes are viral hepatitis, cholestasis, alcoholic, nonalcoholic fatty, and autoimmune diseases of the liver that lead to chronic liver damage [1,2]. The main pathological characteristic of hepatic fibrosis is excessive deposition of extracellular matrix (ECM) in the perisinusoidal space. Hepatic fibrosis can progress to cirrhosis, liver failure, and even liver cancer [3], but it can be reversed to some degree [4,5]. Current drug therapies for hepatic fibrosis are at the exploratory stage of development. No anti-fibrotic therapies can prevent the development of hepatic fibrosis, and therapeutic agents for hepatic fibrosis are urgently needed.

The therapeutic effects of Traditional Chinese medicine (TCM) on diseases have been acknowledged, even though most of their molecular mechanisms remain unknown. Some TCMs have unique anti-fibrotic advantages, such as the ability to suppress liver inflammation, improve hepatic blood flow, and promote liver regeneration [[6], [7], [8]]. We previously confirmed that Yi-Qi-Jian-Pi Formula (YQJPF) comprising Huangqi (*Astragalus propinquus*), Taizishen (*Pseudostellaria Radix*), Baizhu (*Atractylodis Macrocephalae Rhizoma*), Chenpi (*Pericarpium Citri Reticulatae*), Danggui (Radix Angelica Sinensis), Fulin (*Sclerotium Poriae Cocos*), Huangqin (*Scutellaria baicalensis Georgi*), and Gancao (*Radix Glycyrrhizae*) can modulate the PI3K/AKT signaling pathway to attenuate liver failure by suppressing hypoxic injury and apoptosis [9]. We further determined that YQJPF can suppress RIPK1/RIPK3-complex-dependent necroptosis of hepatocytes *via* ROS signaling and attenuated liver damage [10]. In fact, a single Chinese medicine component has a certain anti-liver fibrosis effect. Astragalus propinquus can alleviate dimethylnitrosamine -induced liver fibrosis by regulating the bile acid metabolism enzyme. We also previously investigated the synergistic mechanism of these effective components of YQJPF against hepatic fibrosis using network pharmacology, which might provide a basis for the preparation of this TCM compound for treating hepatic fibrosis [9].

Here, we developed a mouse model induced by carbon tetrachloride (CCl_4_) that replicates some of the features of clinical hepatic fibrosis and assessed the features of disease. We further explored the anti-hepatic fibrosis effects and potential mechanisms of YQJPF. Our findings provide a theoretical basis for applying YQJPF in clinical translational medicine.

## 2 Materials and methods

### 2.1 Animals

Sixty male C57BL/6J mice (Zhuhai Baicutong Biotechnology Co. Ltd., Beijing, China) were housed with laboratory chow and tap water at 22 °C ± 2 °C under 55% ± 5% humidity and a 12-h light–dark cycle. All mice were provided care that complied with the National Institutes of Health (Bethesda, MD, USA) guidelines. The Experimental Animal Ethics Committee at Guangzhou University of Chinese Medicine approved the experimental design (Approval No: 20220420004).

### 2.2 Animal models and treatment

The mice were adapted for one week then randomly assigned to blank control, hepatic fibrosis model, silymarin treatment (positively treated), and low-, medium- and high-dose YQJPF (7.5, 15, and 30 g/kg, respectively). The model, positive, and YQJPF groups received intragastric administrations of CCl_4_ (Tianjin Damao Chemical Reagent Factory, Tianjin, China) for 6 weeks at 3 mL/kg (in 25% diluted olive oil). The controls were administered with an equal volume of olive oil. The mice in the positive treatment group received intragastric silymarin (75 mg/kg) (Jiangsu Zhongxing Pharmaceutical Co., Ltd., Jiangsu, China). Mice in the YQJPF groups were intragastrically administered for 6 weeks (shown in below). The quality of YQJPF was controlled as we previously described [10]. Yi-Qi-Jian-Pi Prescription: Astragalus 30g, Radix pseudostellariae 30g, Angelica 10g, Ligustrum 10g, Poria cocos 10g, Stir-fried atractyloides 30g, Tangerine peel 10g, wine Baicalensis 10g, Roasted licorice 3g.

**Figure:**
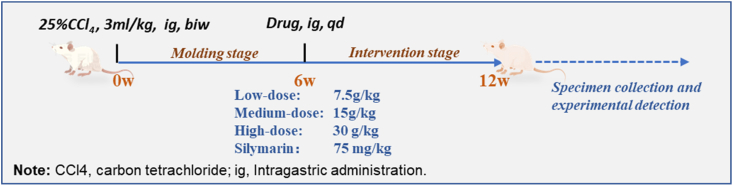

### 2.3 Biochemical parameters and liver function tests

After 6 weeks of drug intervention, blood samples were taken for further study. Levels of alanine aminotransferase (ALT), aspartate aminotransferase (AST), and albumin (ALB) were assayed using kits (Nanjing Jiancheng, Nanjing, China) and prothrombin activity (PTA) was determined using PTA ELISA kits (MultiSciences, Hangzhou, China). All assays proceeded as described by the manufacturers.

### 2.4 Hematoxylin-eosin, masson trichrome and sirius red staining

Mouse liver tissues removed after surgery were rinsed with cold PBS, fixed with 4% paraformaldehyde (Servicebio Technology Co., Ltd., Wuhan, China) for 48 h, routinely embedded in paraffin, then sectioned for hematoxylin and eosin (HE) staining as described by the manufacturer. The section was also stained with Masson trichrome and Sirius red using kits (Servicebio Technology Co., Ltd.) as described by the manufacturer.

### 2.5 Assays of inflammatory biomarkers and oxidative stress assays

Serum levels of the cytokines interleukin-1β (IL-1β), (IL-6) and tumor necrosis factor-α (TNF-α) were assessed using ELISA kits (MultiSciences, Hangzhou, China) as described by the manufacturer. Serum superoxide dismutase (SOD) activity and malondialdehyde (MDA) concentrations were measured using appropriate kits (Nanjing Jiancheng Bioengineering Institute, Nanjing, China).

### 2.6 Macrophage polarization by flow cytometry

Cells were separated or obtained from liver tissues that were washed three times with cold PBS, stained with specific antibodies for 30 min at 4 °C in darkness, and washed three times with cold PBS again. Thereafter, CD11c, F4/80, CD206, and CD86 were detected using an ACEA Novocyte flow cytometer (Agilent Technologies Inc., Santa Clara, CA, USA).

### 2.7 Quantitative reverse transcription-polymerase chain reaction (qRT-PCR)

Total RNA was extracted from liver tissues using AG RNAex Pro Reagent (AG, Guangzhou, China) as described by the manufacturer. Thereafter, cDNA was synthesized using Evo M-MLV (AG) and amplified by RT-PCR using SYBR® Green Pro Taq HS Premix, specific primers (Table 1), and a CFX96 Real-Time PCR Detection System (Bio-Rad Laboratories Inc., Hercules, CA, USA). The mRNA expression of Mothers against Decapentaplegic (Smad) 3, Smad2, and transforming growth factor-beta 1 (TGF-β1) was calculated using the 2^-△△Ct^ method. The expression of all target genes was normalized to that of the internal control, glyceraldehyde-3-phosphate dehydrogenase *GAPDH*.

**Table 1:** Forward (F) and reverse (R) primers for RT-PCR.

Gene	Direction	Sequences (5′ → 3′)
*Smad3*	F	CACGCAGAACGTGAACACC
	R	GGCAGTAGATAACGTGAGGGA
*TGF-β1*	F	CTCCCGTGGCTTCTAGTGC
	R	GCCTTAGTTTGGACAGGATCTG
*Smad2*	F	ATGTCGTCCATCTTGCCATTC
	R	AACCGTCCTGTTTTCTTTAGCTT
*GAPDH*	F	AATTGAGCCCGCAGCCTCCC
	R	AATTGAGCCCGCAGCCTCCC

### 2.8 Immunohistochemical staining

Liver samples fixed in 10% formalin, embedded in paraffin, and cut into 4 μm-thick sections were processed as described by the respective manufacturers. Recombinant Suppressor of Mothers against Decapentaplegic family member 3 (Smad3) (Servicebio Technology Co., Ltd.) and TGF-β (Abcam) antibodies were diluted at 1:500 and 1:200, respectively. The color was developed using 3, 3′-diaminobenzidine (DAB), and hematoxylin counterstaining. The sections were then assessed by light microscopy.

### 2.9 Network pharmacology analysis of Chinese herbal compound

Nine herbs (Radix stellariae Radix, poria cocos, Radix scutellariae, Radix Tangerine peel, Radix atrictyloides, Fructus ligustrum, Radix angelica, Radix glycyrrhizae) of Yi-Qi-Jian-Pi formula were used as search words in the TCMSP (Traditional Chinese Medicine Systems Pharmacology Database and Analysis Platform, https://old.tcmsp-e.com/tcmsp.php). Therefore, Oral absorption rate (OB) ≥30%, Drug-likeness (DL) ≥0.18, and Half-life period (HL) ≥4 were selected as screening criteria to obtain the main components. Then, we predicted the effect of traditional Chinese medicine composition targets by Swiss Target Prediction database (http://www.swisstargetprediction.ch/). Next, we screened out potential targets for liver fibrosis in the OMIM database (https://www.omim.org/) and DisGeNET database (https://www.disgenet.org/) and Gene Card (https://www.genecards.org/). A common target protein-protein interaction (PPI) network was constructed on the String platform and then Network Analyzer module in Cytoscape software is applied to analyze the file information. Finally GO-BP enrichment analysis the selected targets were performed by Metascape (http://metascape.org/) and the selected target protein information was imported into the DAVID (http://david.abcc.ncifcrf.gov) to get the pathways.

### 2.10 Metagenomics and metabolomics detection

Metagenomic and metabolomic detection were was tested and analyzed by Shenzhen BGI Co., LTD. The metagenomics was sequenced on the MGISEQ 2000 platform. Non-targeted metabolomics detection by Liquid chromatography combined with tandem mass spectrometry (LC-MS/MS).

### 2.11 Statistical analysis

Data were analyzed using IBM® SPSS® 24 (IBM Corp., Armonk, NY, USA). All data are expressed as means ± SD. Two or more groups were compared by one-way ANOVA and least significant differences (LSDs) were calculated. ANOVA-Dunnett's T3 method used when no homogeneity was detected. Values with P < 0.05 were considered statistically significant.

## 3 Results

### 3.1 General status of mice

**Fig. 1:**
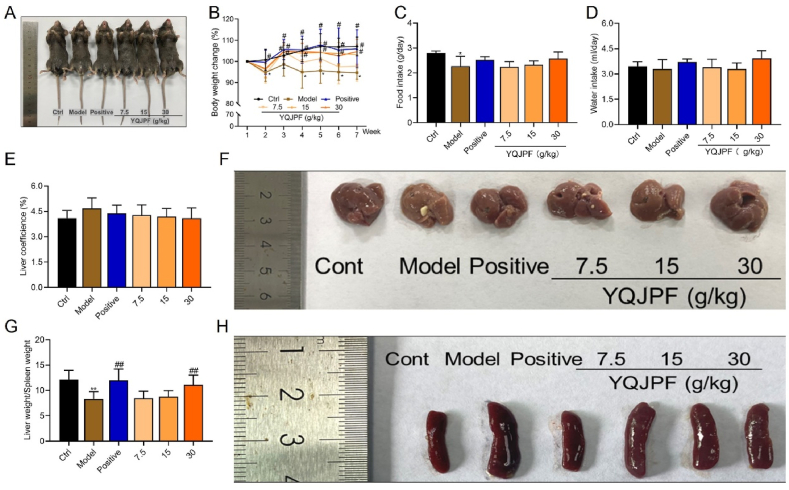
General status of mice. **(A)** Representative images of mice. **(B)** Changes in mouse body weight. Food intake **(C)**, water intake **(D)**, liver coefficients **(E)**, representative gross appearance of liver **(F)**, total liver to spleen ratio **(G)** and representative gross appearance of spleens **(H)** in all mice (*P < 0.05, **P < 0.01 *vs.* control; ^#^P < 0.05, ^##^P < 0.01 *vs*. model).

Body weight significantly decreased in the model and low-dose YQJPF groups. In contrast, body weight significantly increased in the medium, high-dose and positive groups compared with the model mice (P < 0.05, Fig. 1A and B). Significantly less food was consumed by the model, than by control mice, whereas the positive and YQJPF groups consumed slightly more than the model mice (Fig. 1C). Water intake and liver coefficients did not significantly differ between the groups (Fig. 1D‒F). However, inflammatory cell infiltration in the mucosa and submucosa was obvious in the model mice, which had a significantly lower total liver-to-spleen ratio than the controls (P < 0.01). The total liver-to-spleen ratio was substantially increased in the positive and high-dose YQJPF groups compared with the model mice (P < 0.01, Fig. 1G and H).

### 3.2 Liver function was improved and hepatic fibrosis was reversed by YQJPF

**Fig. 2:**
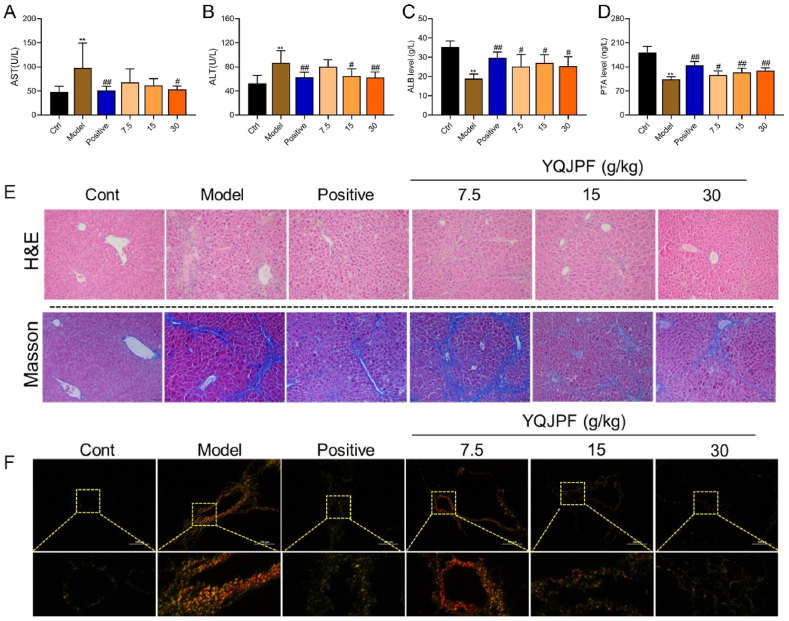
**Hepatic fibrosis was reversed and liver function was improved by YQJPF.** Serum levels of AST **(A)**, ALT **(B)**, ALB **(C)** and PTA **(D)**. Representative images of liver tissues visualized by HE, Masson **(E)** and Sirius Red **(F)** staining (*P < 0.05, ^†^P < 0.01 *vs.* control; *P < 0.05, **P < 0.01 *vs*. model). ALB, albumin; ALT, alanine aminotransferase; AST, aspartate aminotransferase; PTA, prothrombin activity. (For interpretation of the references to color in this figure legend, the reader is referred to the Web version of this article.)

We investigated whether YQJPF could restore the liver function and structure in the mouse models of hepatic fibrosis. Fig. 2A‒D shows significantly increased AST and ALT and decreased ALB and PTA in the models compared with the control group P < 0.01 for all). Staining with HE revealed normal liver architecture including distinct hepatocytes, sinusoidal spaces, and clear central veins in the liver tissues of control mice. The lobular structure was destroyed in livers from the model and low-dose groups, and hepatocyte necrosis was more extensive. In contrast, liver tissues from positive, medium- and high-dose YQJPF mice were relatively normal, with clear lobular structures, orderly hepatic cords, and no obvious pseudo-lobules (Fig. 2E). Masson trichrome and Sirius red staining also showed the same trend with more hepatic fibrosis in the model and low-dose YQJPF groups. Hepatic fibrosis was significantly less extensive in the positive-, medium-, and high-dose, than that in the model mice (Fig. 2E and F).

### 3.3 Inflammation was inhibited and anti-oxidation activity was increased by YQJPF

**Fig. 3:**
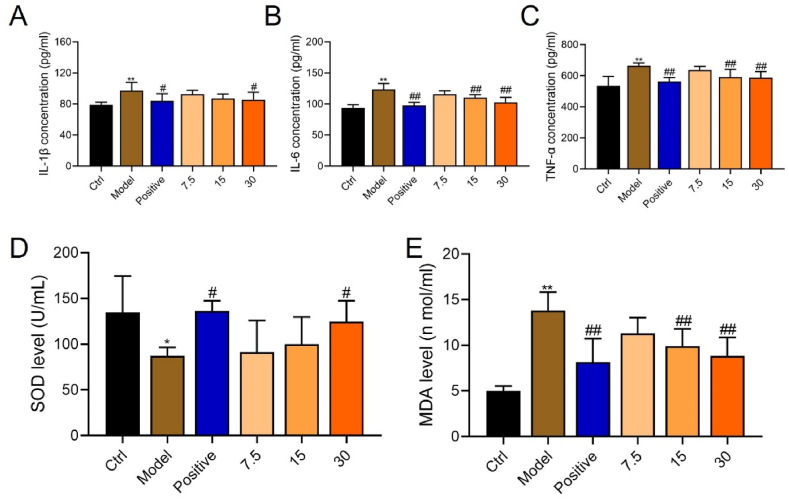
Inflammation was inhibited and oxidation activity was increased by YQJPF. Serum concentrations of IL-1β **(A)**, IL-6 **(B)** and TNF-α **(C)** among groups. Activity of SOD **(D)** and MDA concentration **(E)** were measured using ELISA kits (*P < 0.05, **P < 0.01 *vs.* control; ^#^P < 0.05, ^##^P < 0.01 *vs*. model). IL-1β, interleukin-1β; IL-6, interleukin-6; MDA, malondialdehyde; SOD, superoxide dismutase; TNF-α, tumor necrosis factor-α.

The inflammatory response and DNA damage induced by oxidative stress play important roles in the development and progression of hepatic fibrosis. We measured the inflammatory cytokines IL-1β, IL-6, and TNF-α and the oxidative stress indicators SOD and MDA. Fig. 3A‒C shows significantly increased levels of IL-1β, IL-6, and TNF-α (P < 0.05). However, high-dose YQJPF significantly reduced, whereas the low dose did not significant influence the levels of these cytokines. The medium-dose of YQJPF reduced IL-6 and TNF-α levels.

Oxidative stress is an established major cause of hepatic fibrosis. Therefore, we assessed SOD activity and MDA contents. We found significantly decreased SOD (P < 0.05) and significantly increased MDA levels in the model, compared with control mice (P < 0.001, Fig. 3D and E). High-dose YQJOF significantly increased SOD activity and decreased MDA levels (P < 0.05 for both), and medium-dose YQJPF also reduced MDA levels compared with the model mice (P < 0.01 for both).

### 3.4 M2 macrophage polarization was enhanced by YQJPF

**Fig. 4:**
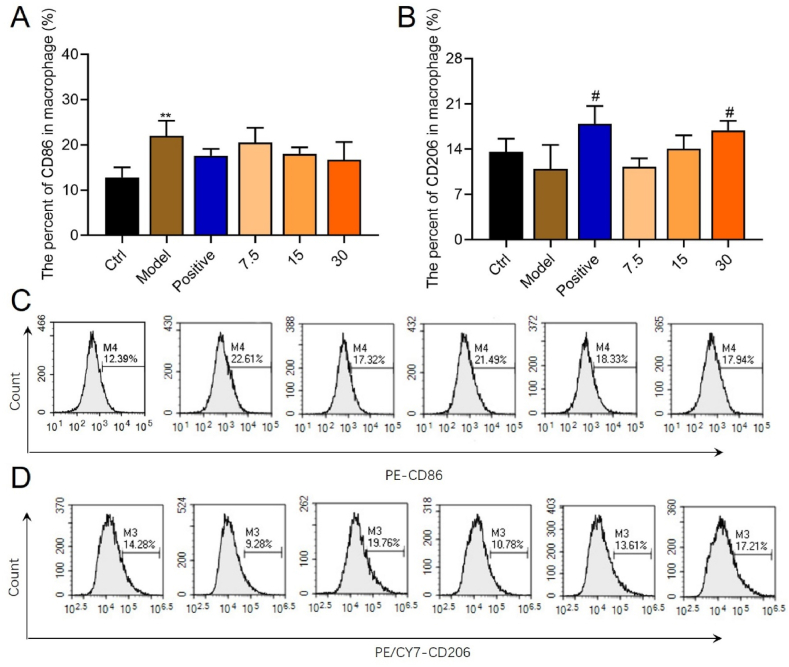
YQJPF enhances M2 macrophage polarization.Ratios (%) of CD86 **(A)** and CD206 **(B)** in macrophages. Representative flow cytometry plots of CD86 **(C)** and CD206 **(D)** (*P < 0.05, **P < 0.05 vs control; **P < 0.01 *vs.* model).

Macrophage polarization plays an important role in the progression of hepatic fibrosis. Therefore, we examined macrophage polarization using flow cytometry (Fig. 4A‒D). The results revealed significantly decreased M1 macrophages (CD86) in the model mice (P < 0.01, Fig. 4A and C) and slightly, but not significantly increased M2 macrophages (CD206) compared with control mice (P > 0.05, Fig. 4B and D). M2 macrophages progressively, dose-dependently, and significantly increased with increasing doses of YQJPF compared with the model mice (Fig. 4B).

### 3.5 Network pharmacology revealed the potential target of YQJPF in treating hepatic fibrosis

**Fig. 5:**
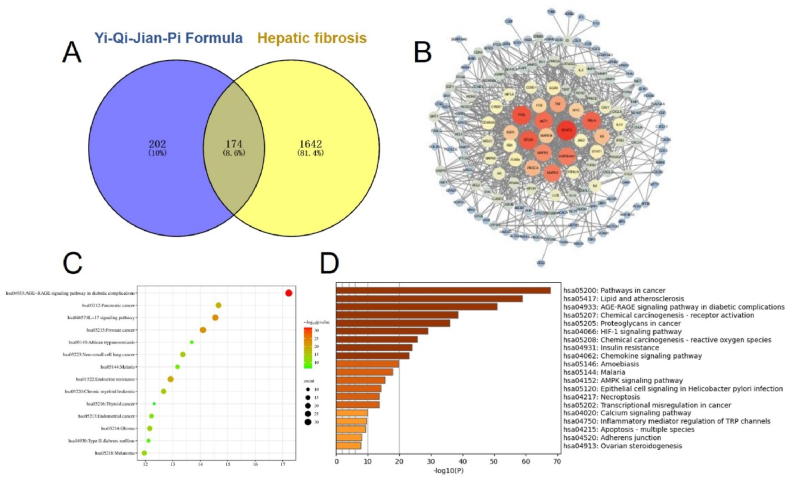
Network pharmacology revealed the potential target of YQJPF in treating hepatic fibrosis (A) Venn diagram of the common targets of YQJPF and liver fibrosis; **(B)** PPI network of potential targets of YQJPF in the treatment of liver fibrosis; **(C)** KEGG pathway analysis of genes related to the anti-fibrosis treatment of YQJPF; **(D)** Enrichment analysis of GO-BP biological function in the treatment of liver fibrosis with YQJPF.

A total of 174 targets were obtained after the intersection of the targets of YQJPF and hepatic fibrosis (Fig. 5A). The PPI network map of 174 common targets was drawn by String (Fig. 5B). A total of 61 hub genes (Degree10 or above) were obtained, including signal transducer and activator of transcription 3(STAT3), tumor protein 53 (TP53), serine/threonine-protein kinase (AKT1), tumor necrosis factor (TNF), phosphoinositide 3 kinase catalytic alpha polypeptide (PIK3CA), peroxisome proliferators-activated receptors (PPARs), Myeloid cell leukemia-1 (MCL1) and transforming growth factor-β (TGF-β) and so on. Subsequently, KEGG pathway analysis (Fig. 5C) and bio-functional enrichment analysis (Fig. 5D) were performed on the targets respectively.

### 3.6 Transforming growth factor beta/Smad3 signaling was inhibited by YQJPF

**Fig. 6:**
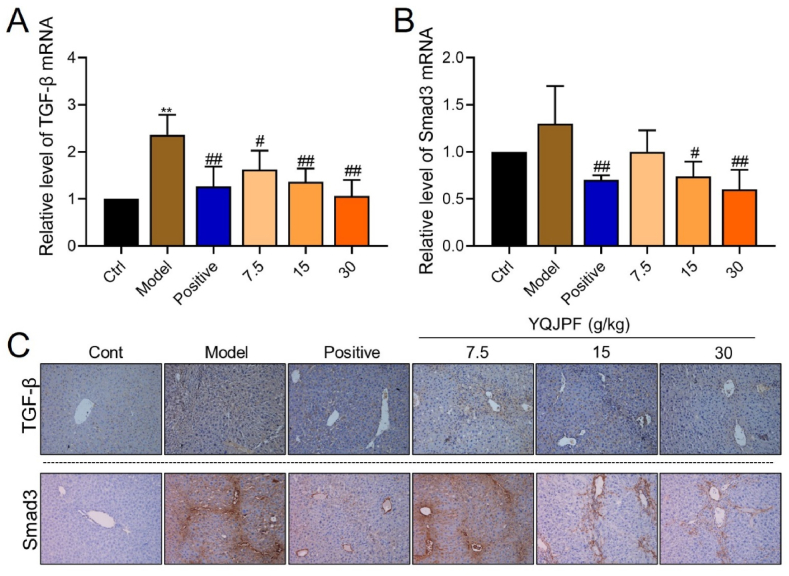
Transforming growth factor beta/Smad3 signaling was inhibited by YQJPF.Relative levels of TGF-β mRNA **(A)** and Smad3 mRNA **(B)** in liver tissues. Representative images of immunohistochemical staining for TGF-β **(C)** and Smad3 **(D)** proteins.

Transforming growth factor beta acts *via* canonical signaling that involves the activation of Smad3 by the TGF-β receptor, followed by the induction of hepatic fibrosis. We found significantly increased TGF-β mRNA and protein expression in liver tissues from the model, compared with control mice (P < 0.01; Fig. 6A and C). We also found significantly increased Smad3 protein and slightly, but not significantly increased Smad3 mRNA in the model mice (Fig. 6C, bottom). However, high-, medium- and low-YQJPF doses inhibited TGF-β expression (P < 0.05, P < 0.01, and P < 0.01, respectively, Fig. 6A), whereas medium- and high-doses of YQJPF reduced Smad3 expression compared with model mice (P < 0.05 and P < 0.01, respectively; Fig. 5B).

### 3.7 YQJPF regulates the structure and abundance of intestinal flora

We analyzed the differences in gut microbiota among Control, Model, and high-dose YQJPF and positive drug groups by metagenomics. The α-diversity and β-diversity of species among the groups were significantly different (See Fig. S1 for details), and the distribution of intestinal flora in each group was analyzed at the phylum and genus levels (Fig. S2). Further analysis showed that the most significant differences between the model group and the control group were Trichomonascaceae, Morganellaceae, Campylobacteraceae, Streptococcaceae, Glomerellacea, etc. (Table 2). The most obvious Top5 bacterial families between YQJPF group and model group were Deferribacteraceae, Enterobacteriaceae, Chroococcaceae, Nautiliaceae and Enterococcacea (Table 3). YQJPF could reduce the expression and abundance of Muribaculum, Lachnoclostridium and Acutalibacter, and increase the abundance of Bacteroides and Brevibacterium in the model mice. Taken together, these results suggest that YQJPF can improve the disordered structure and abundance of bacterial flora in mice with liver fibrosis.

**Table 2:** Top30 different bacteriaceae between model group and control group.

Category	P Value	L_95% CI	R_95% CI	FDR	Meanin All	Meanin C	Meanin M	M vs C
Trichomonascaceae	2.07E-04	−0.0790	−0.0243	0.0632	0.0490	0.0947	0.0033	Down
Morganellaceae	5.83E-04	−1.4453	−0.3820	0.0632	0.7124	1.3111	0.1138	Down
Campylobacteraceae	9.22E-04	0.1263	0.5796	0.0632	0.2344	0.0661	0.4027	Up
Streptococcaceae	1.00E-03	0.1283	0.4659	0.0632	0.2764	0.1125	0.4403	Up
Glomerellaceae	2.83E-03	−1.3673	−0.1199	0.0920	0.7832	1.3058	0.2605	Down
Pyriculariaceae	2.83E-03	−0.6903	−0.1160	0.0920	0.4293	0.7343	0.1243	Down
Chaetomiaceae	3.61E-03	−0.2024	−0.0467	0.0920	0.1272	0.2081	0.0463	Down
Paenibacillaceae	5.43E-03	0.1089	0.4210	0.0920	0.2432	0.1179	0.3685	Up
Enterococcaceae	5.78E-03	0.0851	0.3939	0.0920	0.3973	0.2971	0.4975	Up
Sordariaceae	7.28E-03	−0.2370	−0.0356	0.1020	0.1673	0.2692	0.0654	Down
Rhizobiaceae	9.67E-03	0.0136	0.1391	0.1137	0.0779	0.0383	0.1174	Up
Saccharomycetaceae	1.13E-02	−2.7014	−0.2684	0.1137	1.5958	2.5912	0.6004	Down
Debaryomycetaceae	1.13E-02	−0.4117	−0.0284	0.1137	0.2339	0.3763	0.0914	Down
Sclerotiniaceae	1.40E-02	−1.8258	−0.1139	0.1137	1.1096	1.7893	0.4299	Down
Dipodascaceae	1.40E-02	−0.1191	−0.0058	0.1137	0.0741	0.1196	0.0287	Down
Aspergillaceae	1.73E-02	−0.1836	−0.0160	0.1186	0.1246	0.1945	0.0547	Down
Flavobacteriaceae	2.02E-02	0.0293	0.1596	0.1292	0.0920	0.0432	0.1408	Up
Peptostreptococcaceae	2.11E-02	0.0608	0.6652	0.1331	0.8884	0.6905	1.0862	Up
Burkholderiaceae	2.39E-02	0.0000	0.1557	0.1355	0.0721	0.0340	0.1101	Up
Oscillospiraceae	2.57E-02	0.2523	3.0959	0.1355	3.3497	2.5607	4.1386	Up
Bifidobacteriaceae	2.57E-02	0.0475	0.3967	0.1355	0.4553	0.3856	0.5250	Up
Bacillaceae	2.60E-02	0.0000	0.4082	0.1355	0.1635	0.0578	0.2693	Up
Geobacteraceae	3.20E-02	0.0046	0.1124	0.1355	0.0678	0.0379	0.0978	Up
Rhodobacteraceae	3.57E-02	0.0000	0.1674	0.1355	0.0921	0.0487	0.1354	Up
Lachnospiraceae	3.76E-02	0.3097	7.5398	0.1355	9.4121	7.4930	11.3311	Up
Ruminococcaceae	3.76E-02	0.2676	3.7838	0.1355	7.0814	6.2622	7.9005	Up
Alcanivoracaceae	3.76E-02	−0.1875	−0.0037	0.1355	0.1440	0.2166	0.0714	Down
Desulfobacteraceae	4.35E-02	0.0000	0.0722	0.1483	0.0465	0.0296	0.0634	Down
Hymenobacteraceae	4.43E-02	0.0168	0.2095	0.1497	0.1329	0.0882	0.1775	Down
Actinomycetaceae	4.89E-02	0.0000	0.0879	0.1580	0.0502	0.0306	0.0698	Down

**Table 3:** Differences of bacteria between YQJPF group and model group.

Category	P Value	L_95% CI	R_95% CI	FDR	Meanin All	Meanin M	Meanin YH	YH vs M
Deferribacteraceae	2.62E-03	−0.0835	−0.0240	1	0.0398	0.0641	0.0155	Down
Enterobacteriaceae	1.71E-02	−0.5432	−0.0698	1	0.5147	0.6655	0.3640	Down
Chroococcaceae	1.85E-02	−0.0140	0.0000	1	0.0069	0.0115	0.0023	Down
Nautiliaceae	2.12E-02	−0.0187	0.0000	1	0.0094	0.0142	0.0045	Down
Enterococcaceae	2.57E-02	−0.3560	−0.0377	1	0.4009	0.4975	0.3042	Down
Calotrichaceae	3.50E-02	−0.0129	0.0000	1	0.0025	0.0051	0.0000	Down
Gallionellaceae	3.50E-02	−0.0032	0.0000	1	0.0015	0.0030	0.0000	Down
Prochloraceae	4.07E-02	−0.0135	0.0000	1	0.0075	0.0112	0.0039	Down
Campylobacteraceae	4.51E-02	−0.4842	−0.0254	1	0.2940	0.4027	0.1854	Down

### 3.8 YQJPF regulates the composition of intestinal metabolites

**Fig. 7:**
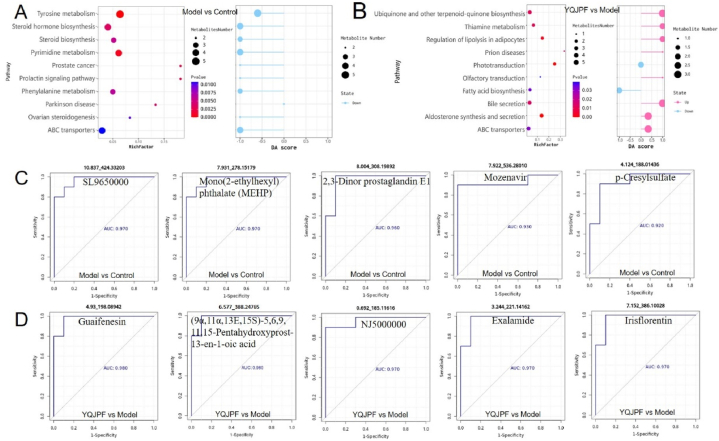
YQJPF regulates the structure and abundance of intestinal flora. (A) Differential metabolic pathways and pathway scores of Top10 between the model group and the control group; (B) Differential metabolic pathways and pathway scores of Top10 between YQJPF group and model group; On the left figure, the RichFactor on the X-axis represents the number of differential metabolites/all metabolites in the pathway. The dot size represents the number of differential metabolites in this Pathway. On the right, the Y-axis represents the name of the metabolic pathway, and the X-axis coordinates represent the differential abundance score (DAscore). DAscore is the global change of all metabolites in a metabolic pathway. A score of 1 indicates an up-regulation trend in the expression of all annotated differential metabolites in the pathway, and a score of negative 1 indicates a down-regulation trend in the expression of all annotated differential metabolites in the pathway. The length of the line segment represents the absolute value of DAscore, and the size of the dot at the end of the line segment represents the number of metabolites in the pathway. The larger the dot, the greater the number of metabolites. (C) Top5 ROC map of differential metabolites between model group and control group; (D) ROC chart of Top5 differential metabolites between YQJPF group and model group; The abscissa is 1-speciﬁcity and the ordinate is sensitivity. The area under the line is the AUC value. A larger AUC value indicates a more suitable metabolite as a biomarker.

After obtaining differential metabolites through non-targeted metabolomics, we divided the four groups into pairwise comparison groups based on different analysis purposes. Fig. 7A and B showed the differential metabolic pathways and pathway score maps between the model group and the control group, and between YQJPF and the model group, respectively. The results suggested that the 10 most significantly different metabolic pathways between the model group and the control group were Tyrosine metabolism, Pyrimidine metabolism, Prolactin signaling pathway and Prostate cancer, Steroid hormone biosynthesis, Parkinson disease, Steroid biosynthesis, Phenylalanine metabolism, Ovarian steroidogenesis and ABC transporters, and Top10 pathway were all down-regulated in the model group compared with the control group. Aldosterone synthesis and secretion, Phototransduction, Regulation of lipolysis in adipocytes, Thiamine metabolism, Ubiquinone and other terpenoid-quinone biosynthesis, Bile secretion, Prion diseases, ABC transporters, Fatty acid biosynthesis were the most significantly different pathways between YQJPF group and model group. And fatty acid biosynthesis and light conduction were up-regulated, and the other 8 metabolic pathways were down-regulated in the YQJPF group.

Then, based on different comparison groups, ROC curve analysis was performed on the differential metabolites to screen potential biomarkers. Fig. 7C shows the ROC curve of Top5 AUC value of differential metabolites between the model group and the control group: SL9650000 (AUC = 0.97), Mono(2-ethylhexyl) phthalate (MEHP) (AUC = 0.97), 2, 3-dinor prostaglandin E1 (AUC = 0.96), Mozenavir (AUC = 0.93) and *p*-Cresylsulfate (AUC = 0.92); Fig. 7D shows the ROC curve of Top5 AUC value of differential metabolites between YQJPF group and model group. Guaifenesin (AUC = 0.98), (9α,11α,13E,15S)-5,6,9,11, 15-pentahydroxyprost-13-en-1-OIC acid (AUC = 0.98), NJ5000000 (AUC = 0.97), Exalamide (AUC = 0.97), and Irisflorentin (AUC = 0.97).

### 3.9 YQJPF may play the anti-fibrosis role by regulating metabolites of intestinal flora

**Fig. 8:**
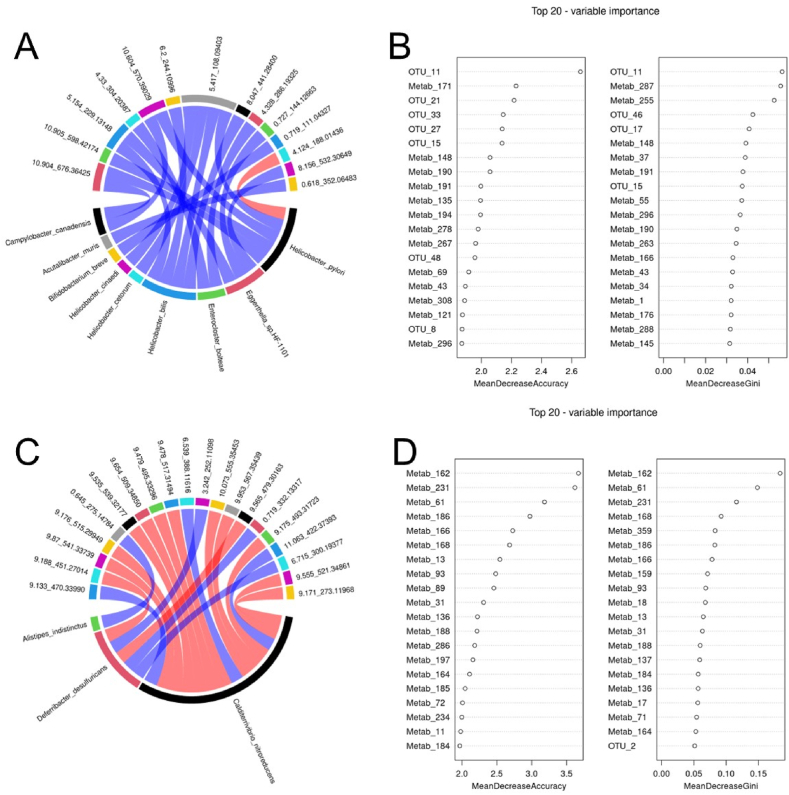
YQJPF regulates the composition of intestinal metabolites (A) The Top20 species and metabolites in the model group and the control group were highly correlated with each other; (B) The random forest analysis importance scores of different species and different metabolites in the model group and the control group are species or metabolites on the edge of the circle of the diagram and the connecting lines in the circle represent the correlation between species and metabolites, red is positive correlation, blue is negative correlation; (C) The correlation diagram of Top20 species and metabolites between YQJPF group and model group; (D) Random forest analysis importance scores of different species and metabolites in YQJPF group and model group. (For interpretation of the references to color in this figure legend, the reader is referred to the Web version of this article.)

We further performed Spearman correlation analysis for differential species and differential metabolites. As shown in Fig. 8A, this chord diagram shows a pair of highly correlated Top20 relationships between the model group and the control group, involving 9 differential bacterial species and 14 differential metabolites, and their specific correlations are shown in Table S1. After screening the above 20 regulatory relationship pairs as potential biomarkers, we screened two groups of biomarkers with the best effect by random forest plots. Fig. 8B shows the species/metabolite scores for top20, where, OTU_11 (Bifidobacterium_breve), OTU_15 (Campylobacter_ canadensis), Metab_148 (N6 - threonylcarbamoyladenosine) and Metab_191 (Loxopr ofen) were ranked in the Top10 with the highest scores of MeanDecreaseAccuracy and MeanDecreaseGini, indicating that they were the most potential biomarkers.

In addition, we furtherly analyzed the correlation of the differential flora and differential metabolites between the YQJPF group and the model group, as shown in Fig. 8C. The chord chart shows the Top20 highly correlated pair between the YQJPF group and the model group, involving 3 differential bacteria species and 20 differential metabolites, and the specific correlation is shown in Table S2. We further screened the most significant biomarkers of bacteria and metabolites through random forest plots (Fig. 8D), and compared Top20 differential metabolites or the difference pairs with high correlation between bacteria and Top20, screened the repeated terms, and obtained the three most significant biomarkers of metabolites were as follows: 10.133_470.33990 (18-β-glycyrrhetinic acid, C30H46O4), 10.073_555.35453 (O-(Hydroxy{(2R)-2-hydroxy-3-[(2-methoxynonadecyl)oxy]propoxy}phosphoryl)-l-serine), 6.715_300. 19377 (13, 14-dihydro-15-keto-tetranor prostaglandin F1a, C16H28O5). Among them, 18β-glycyrrhetinic acid (18β-GA) is a natural compound widely present in Chinese herbs such as licorice, which is used to treat a variety of liver diseases. YQJPF can inhibit the abundance of Calditerrivibrio_nitroreducens and increase the expression of 18β-glycyrrhetinic acid, and there is a significant negative correlation between the two. These results suggest that Calditerrivibrio_nitroreducens may reduce the anti-fibrosis effect of Chinese herbs such as licorice by digesing 18β-glycyrrhetinic acid, but this correlation needs to be further verified.

## 4 Discussion

Hepatic fibrosis arises due to chronic liver damage along with the deposition of ECM proteins, which is a common feature of most types of chronic liver diseases. Accumulated myofibroblastic cells and increased ECM production are closely associated with healing the damaged liver [11]. Hepatic fibrosis induced by CCl_4_ in mice is a widely accepted experimental model, which mirrors in many ways the human disease pattern associated with toxic injury, as stellate cell activation and key matrix components and this model have been demonstrated in the pathogenesis of this model. The process of early hepatic fibrosis can generally be easily reversed, but hepatic fibrosis is likely to further progress to cirrhosis if damaging factors are not removed [12,13]. Timely removal of irritants and reasonable intervention can reverse hepatic fibrosis [14]. Therefore, an in-depth analysis of the mechanism and the identification of effective drugs for hepatic fibrosis are of great significance for treating hepatic fibrosis.

We investigated the effects of three doses of YQJPF on inflammation, oxidative stress, macrophage polarization, and the regulation of related signaling pathways in mouse models with CCl_4_-induced hepatic fibrosis. We found that YQJPF dose-dependently increased body weight, improved liver function, and reversed hepatic fibrosis. Because inflammatory cytokines play crucial roles in the development and progression of hepatic fibrosis [14,15], we quantified IL-1β, IL-6, and TNF-α levels using ELISA kits. Elevated levels of these pro-inflammatory cytokines in the model mice were substantially decreased by YQJPF, particularly at the high dose (Fig. 3A‒C); these results were in line with previous findings [[16], [17], [18]]. Our results also indicated that YQJPF exerted anti-inflammatory effects in our model mice. Oxidative stress plays a predominant pro-fibrotic role in hepatic fibrosis [19,20]. We found elevated MDA levels and reduced SOD activity in serum from model mice. High-dose YQJPF notably decreased the MDA concentration and increased SOD activity. The therapeutic application of antioxidative stress can inhibit and even reverse the progression of hepatic fibrosis [21,22]. Our results indicated that YQJPF contributes to oxidative stress resistance, which might be one mechanism through which YQJPF exerts anti-fibrosis effects.

Heterogeneous macrophages can quickly change their phenotype, activation status, and function in response to signals from the local milieu. Macrophages play important roles in various immune responses and they are broadly classified as having M1 or M2 phenotypes. Macrophage activation with M1/M2 expression contributes to hepatoprotective effects against fibrosis. Macrophages are believed to play crucial roles in host defense, immune regulation, liver regeneration, tissue repair, and immunity. M1 macrophages secrete proinflammatory cytokines and chemokines such as IL-1β, IL-2, IL-6, IL-15, and TNF-α during the early phase of inflammation. When infection or injury is controlled, macrophages initiate M2 polarization and secrete anti-inflammatory molecules, including IL-1 receptor antagonists (IL-1RA) and IL-10. These activities inhibit the production of proinflammatory cytokines and consequently decrease hepatic fibrosis. Here, we found significantly decreased M1 (CD86) and mildly, but not significantly, decreased M2 macrophages in our mouse models of hepatic fibrosis. However, high-dose YQJPF increased the abundance of M2 polarized macrophages, indicating that YQJPF exerts anti-fibrotic effects in the liver by modulating immune system functions.

We screened active compounds and potential targets using network pharmacology and identified TGF-β1 as one of therapeutic targets of YQJPF. TGF-β/Smad3 signaling functions in hepatic fibrosis by activating hepatic stellate cells (HSCs) and ECM accumulation [23,24]. Several Chinese herbal medicines such as Eugenol [25], Xiaochaihu [26], Kushen [27], Α-lipoic acid [28], and Guizhi-Fuling pill [29] inhibit fibrotic diseases, by blocking TGF-β/Smad3 signaling. We also found that YQJPF inhibited TGF-β/Smad3, which might be one of the mechanisms through which YQJPF exerts anti-fibrotic effects in the liver.

Currently, it is believed that the decrease of beneficial bacteria and the increase of potential pathogenic bacteria are a common feature of cirrhosis and liver fibrosis [30,31]. In this study, The association analysis of metagenomic and metabolite data was performed to screen many species and metabolites with potential biomarker significance from different perspectives. The abundance of Calditerrivibrio_ nitroreducens in YQJPF group was 5.1 times lower than that in the model group, while the level of 18β-glycyrrhetinic acid was 37.4 times higher than that in the model group. Correlation analysis suggested that there was a negative correlation between them. 18β-glycyrrhetinic acid is a natural compound widely present in Chinese herbs such as licorice, which is used to treat a variety of liver diseases. YQJPF can inhibit the abundance of Calditerrivibrio_nitroreducens and increase the expression of 18β-glycyrrhetinic acid, and there is a significant negative correlation between these two. These results suggest that Calditerrivibrio_nitroreducens may reduce the anti-fibrosis effect of Chinese herbs such as licorice by digesing 18β-glycyrrhetinic acid which needs to be verified by further experiments.

In conclusion, our findings provide unequivocal support for our initial predictions. We showed that YQJPF attenuated CCl_4_-induced hepatic fibrosis and improved liver function by inhibiting inflammation, suppressing oxidative stress, regulating the immunological response initiated by macrophages, and inhibiting TGF-β/Smad3 signaling. The mechanism may be related to the regulation of intestinal flora homeostasis through multi-target regulation. Most importantly, we confirmed that YQJPF could be included in clinical regimens to treat hepatic fibrosis. Further investigation is required to determine the clinical feasibility of this approach.

## Funding

The work was supported by the Leading Talent Project of 10.13039/501100002949Jiangsu Province Traditional Chinese Medicine (SLJ0216), the Open Project of Jiangsu Key Laboratory of Regional Resource Exploitation and Medicinal Research (LPRK202104).

## Data availability statement

The datasets of Metagenome and metabolome for this study can be found in the “Baidu Netdisk” (https://pan.baidu.com/s/1RfHtmzCnIiedijswDyYyGg?pwd=heli with the extraction code “heli”.

## CRediT authorship contribution statement

**Shiyan Yang:** Writing – original draft, Formal analysis, Data curation. **Yajun Cheng:** Writing – original draft, Formal analysis, Data curation. **Xiaolong Wang:** Data curation, Conceptualization. **Suyang Yue:** Investigation, Formal analysis. **Xi Wang:** Software. **Li Tang:** Software, Resources. **Hailun Li:** Data curation. **Jie Zhang:** Writing – original draft, Software, Data curation. **Qingping Xiong:** Investigation, Conceptualization. **Shanzhong Tan:** Writing – review & editing, Project administration, Funding acquisition.

## Declaration of competing interest

The authors declare that they have no known competing financial interests or personal relationships that could have appeared to influence the work reported in this paper.
