# Supplementary material for: Chinese herbal decoction, Yi-Qi-Jian-Pi formula exerts anti-hepatic fibrosis effects in mouse models of CCl4-induced liver fibrosis
Source: Heliyon. 2024 Feb 22;10(5):e26129. doi: 10.1016/j.heliyon.2024.e26129 (PMC10907526; doi:10.1016/j.heliyon.2024.e26129)
Supplement: Multimedia component 1 [file mmc1.docx]

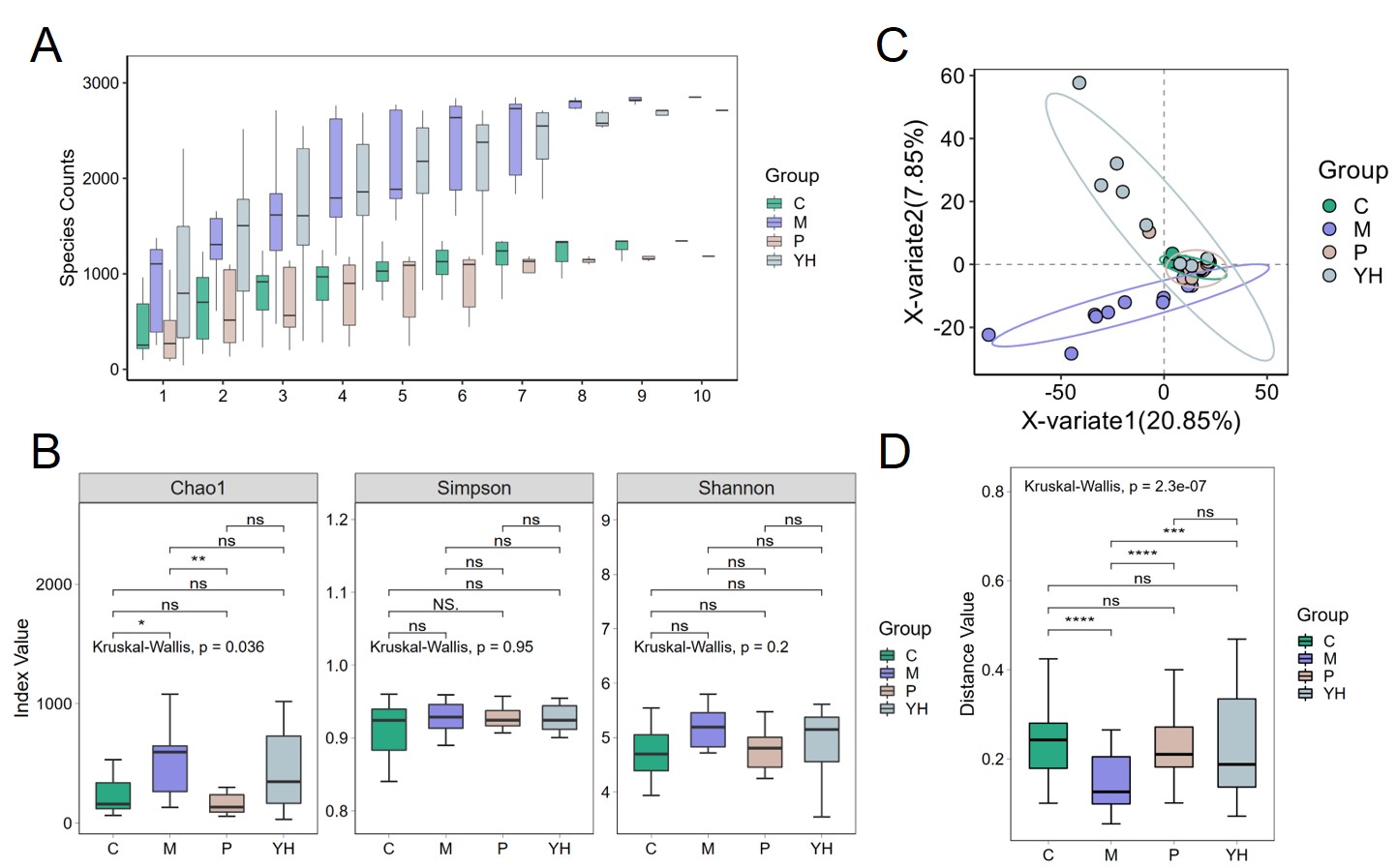


**Figure S1 Alpha-diversity and Beta-diversity of species between groups**

(A) Boxplot of species dilution curves. The abscissa represents the number of samples, the ordinate represents the number of species detected, and the color of the bins represents the different groupings. Each box represents the number of species that can be obtained at the specified number of samples. The top and bottom edges of the box indicate the upper and lower quartile of the distance within the group, the horizontal line in the box indicates the median distance, and the end of the straight line above and below the box indicates the maximum and minimum distance. (B) box plot of α-diversity of species. Each boxplot represents a diversity index, the ordinate is the index value, and the method and result of hypothesis test are marked at the top left of each boxplot. (C) PLS-DA analysis scatter plot. Each point represents a sample, the color of the point represents the group in which the sample is located, the horizontal and vertical coordinates represent the PLS dimension that separates the samples, the value of the axis title bracket is the explanation rate of the dimension to the sample after dimension reduction, and the confidence level of the ellipse confidence interval is 95%. (D) box plot of β-diversity of species. Each bin reflects the distribution of the corresponding distance within the group. A larger median indicates a larger sample distance within the group, and a longer bin length and the length of the line outside the bin indicate a larger distance difference within the group: **** P<0.0001, *** P<0.001, ** P<0.01, * P<0.05, ns represents P> 0.05, NS means P = 1.


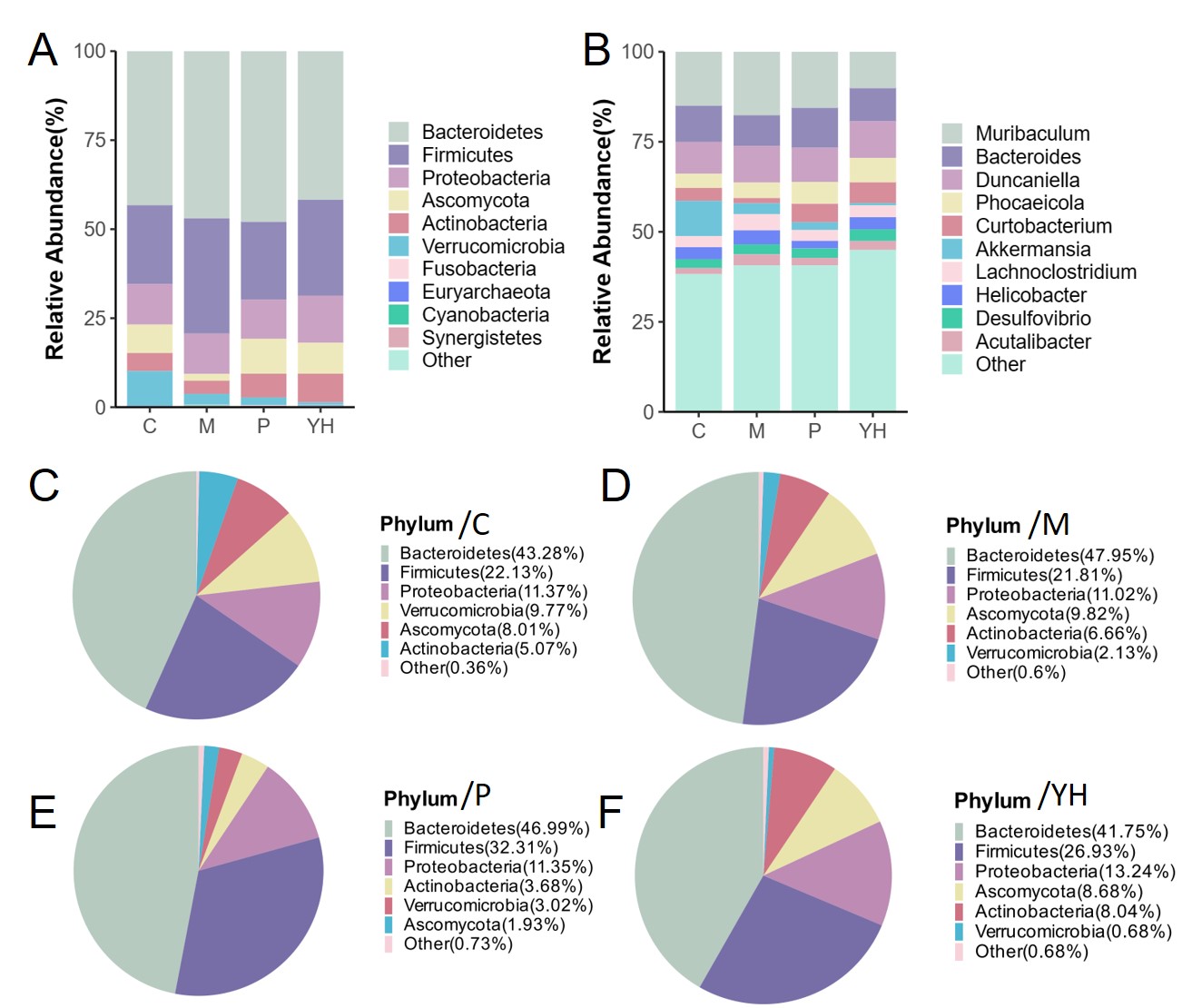


**Figure S2 intestinal flora distribution**

Columnar stacked plots of species abundance at phylum level (A) and genus level (B). The abscissa is sample/group, and the ordinate is relative abundance of species. The color of the column indicates species classification, and the longer the column is, the higher its relative abundance is. Pie chart of genus-level species in control group (C), model group (D), silymarin group (E), and YQJPF group (F). Different sector areas and sector colors indicate different species classifications, and sector center angles indicate the proportion of counterpart species in the total population.
