# Supplementary material for: Chinese herbal decoction, Yi-Qi-Jian-Pi formula exerts anti-hepatic fibrosis effects in mouse models of CCl4-induced liver fibrosis
Source: Heliyon. 2024 Feb 22;10(5):e26129. doi: 10.1016/j.heliyon.2024.e26129 (PMC10907526; doi:10.1016/j.heliyon.2024.e26129)
Supplement: Multimedia component 2 [file mmc2.docx]

**Table S1 Top20 high correlation pairs between model group and control grou**

| Species | Metablites ID | Metabolites name | R | P value |
| --- | --- | --- | --- | --- |
| Helicobacter_pylori | 10.904_676.36425 | Gingerglycolipid A | -0.8082 | 1.63E-05 |
| Helicobacter_pylori | 10.905_598.42174 | [8-Formyl-5-methoxy-2-methyl-2-(4-methyl-2-oxo-3-penten-1-yl)-3,4-dihydro-2H-chromen-7-yl]methyl stearate | -0.8005 | 2.25E-05 |
| Eggerthella_sp.HF-1101 | 5.154_229.13148 | Butenylcarnitine | -0.7959 | 2.71E-05 |
| Helicobacter_pylori | 10.604_570.39029 | [8-Formyl-5-methoxy-2-methyl-2-(4-methyl-2-oxo-3-penten-1-yl)-3,4-dihydro-2H-chromen-7-yl]methyl palmitate | -0.7913 | 3.25E-05 |
| Helicobacter_bilis | 6.2_244.10996 | Osthol | -0.7877 | 3.74E-05 |
| Enterocloster_bolteae | 4.33_304.20387 | Methyldienolone | -0.7925 | 4.18E-05 |
| Helicobacter_cetorum | 5.417_108.09403 | 4-Vinylcyclohexene | -0.7830 | 4.46E-05 |
| Helicobacter_cinaedi | 5.417_108.09403 | 4-Vinylcyclohexene | -0.7815 | 4.73E-05 |
| Helicobacter_bilis | 8.047_441.28400 | 2-Aminoethyl (2R)-2-hydroxy-3-[(2-methoxytetradecyl)oxy]propyl hydrogen phosphate | -0.7807 | 4.87E-05 |
| Helicobacter_bilis | 5.154_229.13148 | Butenylcarnitine | -0.7753 | 5.94E-05 |
| Eggerthella_sp.HF-1101 | 0.727_144.12663 | Acetylcadaverine | -0.7728 | 6.48E-05 |
| Helicobacter_bilis | 5.417_108.09403 | 4-Vinylcyclohexene | -0.7721 | 6.64E-05 |
| Helicobacter_pylori | 5.417_108.09403 | 4-Vinylcyclohexene | -0.7698 | 7.22E-05 |
| Bifidobacterium_breve | 0.719_111.04327 | Cytosine | -0.7682 | 7.62E-05 |
| Enterocloster_bolteae | 4.328_286.19325 | Androstenedione | -0.7774 | 8.09E-05 |
| Eggerthella_sp.HF-1101 | 8.156_532.30649 | 5β-cyprinol sulfate | -0.7652 | 8.48E-05 |
| Helicobacter_pylori | 4.124_188.01436 | p-Cresylsulfate | 0.7652 | 8.48E-05 |
| Campylobacter_canadensis | 10.604_570.39029 | [8-Formyl-5-methoxy-2-methyl-2-(4-methyl-2-oxo-3-penten-1-yl)-3,4-dihydro-2H-chromen-7-yl]methyl palmitate | -0.7638 | 8.89E-05 |
| Campylobacter_canadensis | 10.904_676.36425 | Gingerglycolipid A | -0.7638 | 8.89E-05 |
| Acutalibacter_muris | 0.618_352.06483 | 4-O-(4-Deoxy-beta-L-threo-hex-4-enopyranuronosyl)-beta-D-galactopyranuronic acid | -0.7639 | 0.000134 |

Table S2 Top20 high correlation pairs between YQJPF group and model group

| Species | Metablites ID | Metabolites name | R | P value |
| --- | --- | --- | --- | --- |
| Calditerrivibrio_nitroreducens | 9.133_470.33990 | 18β-glycyrrhetinic acid | -0.81607 | 1.15E-05 |
| Deferribacter_desulfuricans | 9.188_451.27014 | (2R)-3-{[(2-Aminoethoxy)(hydroxy)phosphoryl]oxy}-2-hydroxypropyl (9Z)-9-hexadecenoate | 0.785128 | 4.12E-05 |
| Calditerrivibrio_nitroreducens | 9.87_541.33739 | O-(Hydroxy{(2R)-2-hydroxy-3-[(2-methoxyoctadecyl)oxy]propoxy}phosphoryl)-L-serine | 0.78026 | 4.95E-05 |
| Calditerrivibrio_nitroreducens | 9.176_515.29949 | LysoPC(18:4(6Z,9Z,12Z,15Z)) | 0.77645 | 5.69E-05 |
| Alistipes_indistinctus | 0.645_275.14784 | epsilon-(gamma-Glutamyl)-lysine | -0.77594 | 8.58E-05 |
| Calditerrivibrio_nitroreducens | 9.535_539.32177 | O-{Hydroxy[(2R)-2-hydroxy-3-(nonadecanoyloxy)propoxy]phosphoryl}-L-serine | 0.77264 | 6.53E-05 |
| Calditerrivibrio_nitroreducens | 9.654_509.34850 | 1-heptadecanoyl-sn-glycero-3-phosphocholine | 0.76883 | 7.46E-05 |
| Calditerrivibrio_nitroreducens | 9.479_495.33296 | Lysolecithin | 0.766544 | 8.08E-05 |
| Calditerrivibrio_nitroreducens | 9.478_517.31494 | LysoPC(18:3(9Z,12Z,15Z)) | 0.760449 | 9.95E-05 |
| Calditerrivibrio_nitroreducens | 6.539_388.11616 | 5-Demethylnobiletin | -0.75969 | 1.02E-04 |
| Deferribacter_desulfuricans | 3.242_252.11098 | Ala-Tyr | -0.75747 | 1.10E-04 |
| Calditerrivibrio_nitroreducens | 10.073_555.35453 | O-(Hydroxy{(2R)-2-hydroxy-3-[(2-methoxynonadecyl)oxy]propoxy}phosphoryl)-L-serine | 0.749019 | 1.45E-04 |
| Calditerrivibrio_nitroreducens | 9.953_567.35439 | O-{[(2R)-3-(Henicosanoyloxy)-2-hydroxypropoxy](hydroxy)phosphoryl}-L-serine | 0.745971 | 1.59E-04 |
| Deferribacter_desulfuricans | 9.565_479.30163 | 1-Oleoyl-2-hydroxy-sn-glycero-3-PE | 0.742107 | 1.80E-04 |
| Deferribacter_desulfuricans | 0.719_332.13317 | Zanamivir | -0.74057 | 1.88E-04 |
| Calditerrivibrio_nitroreducens | 9.175_493.31723 | 1-[(9Z)-hexadecenoyl]-sn-glycero-3-phosphocholine | 0.738351 | 2.01E-04 |
| Calditerrivibrio_nitroreducens | 11.063_422.37393 | (5Z,9Z)-2-Methoxy-5,9-hexacosadienoic acid | -0.73759 | 2.06E-04 |
| Deferribacter_desulfuricans | 6.715_300.19377 | 13,14-Dihydro-15-keto-tetranor prostaglandin F1a | -0.7375 | 2.07E-04 |
| Calditerrivibrio_nitroreducens | 9.555_521.34861 | MFCD00133435 | 0.735303 | 2.21E-04 |
| Calditerrivibrio_nitroreducens | 9.171_273.11968 | 6-{[2-(2-Thienyl)ethyl]amino}-5,6,7,8-tetrahydro-1-naphthalenol | 0.734541 | 0.000226 |
